# Electrophysiology of Inhibitory Control in the Context of Emotion Processing in Children With Autism Spectrum Disorder

**DOI:** 10.3389/fnhum.2019.00078

**Published:** 2019-03-12

**Authors:** Justine R. Magnuson, Nicholas A. Peatfield, Shaun D. Fickling, Adonay S. Nunes, Greg Christie, Vasily Vakorin, Ryan C. N. D’Arcy, Urs Ribary, Grace Iarocci, Sylvain Moreno, Sam M. Doesburg

**Affiliations:** ^1^Department of Biomedical Physiology and Kinesiology, Simon Fraser University, Burnaby, BC, Canada; ^2^Behavioural and Cognitive Neuroscience Institute, Simon Fraser University, Burnaby, BC, Canada; ^3^CTF MEG International Services, Vancouver, BC, Canada; ^4^School of Engineering Science, Simon Fraser University, Surrey, BC, Canada; ^5^NeuroTech Laboratory, Surrey Memorial Hospital, Surrey, BC, Canada; ^6^Digital Health Hub, Simon Fraser University, Surrey, BC, Canada; ^7^AGE-WELL National Innovation Hub: Digital Health Circle, Surrey, BC, Canada; ^8^Surrey Memorial Hospital, Health Sciences and Innovation, Surrey, BC, Canada; ^9^Department of Psychology, Simon Fraser University, Burnaby, BC, Canada; ^10^Department Pediatrics and Psychiatry, University of British Columbia, Vancouver, BC, Canada; ^11^BC Children’s Hospital Research Institute, Vancouver, BC, Canada; ^12^Department of School of Interactive Art and Technology, Simon Fraser University, Surrey, BC, Canada

**Keywords:** autism spectrum disorder, electroencephalography, event related potentials, inhibitory control, emotion processing

## Abstract

Autism Spectrum Disorder (ASD) is an increasingly common developmental disorder that affects 1 in 59 children. Despite this high prevalence of ASD, knowledge regarding the biological basis of its associated cognitive difficulties remains scant. In this study, we aimed to identify altered neurophysiological responses underlying inhibitory control and emotion processing difficulties in ASD, together with their associations with age and various domains of cognitive and social function. This was accomplished by assessing electroencephalographic recordings during an emotional go/nogo task alongside parent rating scales of behavior. Event related potential (ERP) N200 component amplitudes were reduced in children with ASD compared to typically developing (TD) children. No group differences were found, however, for task performance, P300 amplitude or latency, or N170 amplitude or latency, suggesting that individuals with ASD may only present conflict monitoring abnormalities, as reflected by the reduced N200 component, compared to TD individuals. Consistent with previous findings, increased age correlated with improved task performance scores and reduced N200 amplitude in the TD group, indicating that as these children develop, their neural systems become more efficient. These associations were not identified in the ASD group. Results also showed significant associations between increased N200 amplitudes and improved executive control abilities and decreased autism traits in TD children only. The newly discovered findings of decreased brain activation in children with ASD, alongside differences in correlations with age compared to TD children, provide a potential neurophysiological indicator of atypical development of inhibitory control mechanisms in these individuals.

## Introduction

Autism spectrum disorder (ASD) is a neurodevelopmental disorder that affects 1 in 59 children, an increase in prevalence of 130% since 2002 (Autism and Developmental Disabilities Monitoring Network Surveillance Year 2010 Principal Investigators, 2014). Individuals with ASD experience a wide range of challenges, including core deficits in social communication and repetitive behaviors and/or restricted interests (American Psychiatric Association, 2013). These individuals also commonly show difficulties in various executive functions including inhibitory control (Hill, 2004; Lopez et al., 2005; Sinzig et al., 2008). Although behavioral scores can be effective for measuring inhibitory control, behavioral studies have drawn inconsistent conclusions regarding these executive functions in individuals with ASD (Geurts et al., 2014). Several studies found reduced behavioral performance (accuracy and/or reaction time) in individuals with ASD for go/nogo tasks, which require inhibition of a prepotent response, compared to typically developing (TD) individuals (Christ et al., 2007; Langen et al., 2012; Xiao et al., 2012), whereas other studies revealed no significant differences between groups with regard to behavioral performance on the go/nogo task (Schmitz et al., 2006; Kana et al., 2007; Sinzig et al., 2008; Lee et al., 2009). Understanding the neurophysiological underpinnings of inhibitory control abilities in ASD may help clarify the inconsistent results obtained in previous behavioral studies.

EEG studies reveal that during the go/nogo task, two well-defined and highly reliable event related potential (ERP) components are consistently elicited: a negative-going component (N200) and a positive-going component (P300) (Falkenstein et al., 1999; Rietdijk et al., 2014). The N200 component peaks at approximately 200–300 ms post-stimulus in adults, and approximately 300–400 ms post-stimulus in children (Espinet et al., 2012; Brydges et al., 2013; Shephard et al., 2014; Vuillier et al., 2016). This component corresponds to detection of novelty, response conflict and error monitoring (Donkers and Van Boxtel, 2004; Folstein and Van Petten, 2008). The P300 component peaks at approximately 300–500 ms post-stimulus in adults, and up to 600 ms post-stimulus in children, and reflects the cognitive inhibitory process related to the actual inhibition of the motor response including selection of responses (Donkers and Van Boxtel, 2004; Folstein and Van Petten, 2008; Brydges et al., 2014; Rietdijk et al., 2014; Shephard et al., 2014; Kompatsiari et al., 2016). The prefrontal cortex and the anterior cingulate cortex (ACC) are involved in inhibitory control and are the primary generators for the P300 and N200 in this context, respectively (Bokura et al., 2001; Rubia et al., 2001). In response to the go/nogo task specifically, the N200 component observed in both the go (N200-go) and nogo (N200-nogo) trials, and the P300 component observed in nogo trials (P300-nogo) have been localized to frontocentral scalp locations, while the P300 component observed in go trials (P300-go) has been localized to parietal scalp positions (Jonkman et al., 2003; Jonkman, 2006; Jia et al., 2017). Correspondingly, an inefficiency in response inhibition reflects an absence or reduction of frontocentral P300-nogo component amplitude (Jonkman et al., 2003), while a deficit in conflict monitoring abilities implies abnormalities in the mechanism involving ACC signaling for increased cognitive control, as reflected in the anterior or frontocentral N200 component (Botvinick et al., 2001; Folstein and Van Petten, 2008).

Few studies have investigated ERP correlates of inhibition in ASD during a go/nogo task, and according to our research, only two studies have investigated N200/P300 effects during a go/nogo task in these individuals (Høyland et al., 2017; Kim et al., 2018). Kim et al. found no significant differences in N200 amplitude on both the go and nogo trials across the ASD and TD groups (Kim et al., 2018). Significantly smaller go/nogo P300 amplitude differences in the ASD group compared to the TD group were reported, however, possibly indicating less efficient response priming of nogo trials in the ASD group. Høyland et al. found no significant difference in P300-go/nogo, N200-go/nogo or N200-effect in individuals with ASD compared to TD individuals (2017). Although few differences were identified across ASD and TD groups in each of these studies, the study by Kim et al. had a small sample size, consisting of 9 children with ASD and 17 TD children, and assessed a younger age group (average age of 5 years old), whereas Høyland et al. assessed only older individuals of 12–21 years of age. Investigating differences in the neural markers of inhibitory control between ASD and TD individuals during the developmental period of 6–12 years of age is essential to understanding the development of this crucial brain process in ASD. Moreover, investigating neurophysiological responses in ASD and their associations with age is particularly important given that other studies have reported a decrease in N200 amplitude with increasing age during this specific period of development in TD children (Johnstone et al., 2005, 2007; Jonkman, 2006).

Inhibitory control is thought to play a role in many cognitive domains, including emotion regulation/recognition (Dennis et al., 2009). Individuals with ASD commonly display processing abnormalities specific to facial expressions of emotion, including reduced and delayed N170 amplitude and latency, respectively, to emotional facial expressions compared to TD individuals (Dawson et al., 2005; Batty et al., 2011; Tye et al., 2014b). Behavioral studies assessing recognition abilities of emotional facial expression in ASD have shown that individuals with ASD are worse at recognizing emotion when face stimuli are presented quickly, and when emotional expression is subtle (Rump et al., 2009). Age has also been shown to have an impact on the differences in emotion processing abilities between ASD and TD individuals, such that children with ASD who are above the age of 12 typically process emotion no differently than TD individuals, however, at 10 years of age, children with ASD are worse than TD individuals at labeling basic prototypic emotional expressions (Capps et al., 1992; Lindner and Rosén, 2006).

Investigating behavioral and neurophysiological responses to an inhibitory control task that involves emotion processing could, therefore, provide important information regarding both the separable functions of these cognitive processes, as well as the relationship between them. More specifically, such a task can assess potential differences in N170 amplitude and latency responses to angry and happy faces across ASD and TD groups, as well as any behavioral differences on inhibitory control performance for angry and/or happy trials across groups. Studies have shown that emotional face stimuli can either (a) interrupt ongoing cognitively controlled tasks, effectively reducing attentional allocation to the given inhibitory control task resulting in reduced task performance scores (Verbruggen and De Houwer, 2007; de Houwer and Tibboel, 2010), or (b) increase salience to the inhibitory control task, increasing the processing speed and possibly performance accuracy of the inhibitory stimuli (Taylor et al., 2018). Determining the direction and degree of interaction between happy face stimuli, angry face stimuli and inhibitory stimuli in children with ASD compared to TD children would, therefore, be highly beneficial.

To our knowledge, this is the first study to have correlated neurophysiological responses to an emotional go/nogo task (N200, P300, and N170 component amplitudes and latencies) in individuals with ASD with age and behavioral scores of executive functions, autism traits, intelligence, and social competence. We hypothesized that children with ASD would have reduced amplitudes and prolonged latencies of the N200, P300, and N170 components compared to TD individuals, suggesting deficits in later-stage processing of the stimuli relating to stimuli categorization, response inhibition, and emotion processing, respectively. Lastly, we hypothesized that neurophysiological responses, which are reduced during inhibitory control in ASD would be correlated with lower response accuracy scores, parent rating scores, IQ scores, and higher autism traits. Identifying a comprehensive understanding of potential inhibitory control abnormalities, alongside the developmental trajectories of these abnormalities in ASD compared to TD individuals holds the potential to serve as a useful tool in targeted treatment efforts.

## Materials and Methods

### Data Collection

Data were collected from multiple children during four single-day summer camps using methods previously developed by our research group (Moreno et al., 2015, 2011). In general, these camps involved multiple research groups running behavioral, and/or neurophysiological examinations to both TD and ASD children. Groups of four to six children were tested simultaneously in a large room and alternated at 1 h intervals, providing approximately 40 min windows for EEG collection from each group of subjects.

### Participants

Participants with ASD had a prior diagnosis of ASD as received by a qualified pediatrician, psychologist or psychiatrist associated with the government-funded ASD assessment network or with a qualified private clinic in British Columbia (BC). All diagnoses of ASD were based on the Diagnostic and Statistical Manual of Mental Disorders (DSM) and confirmed using the Autism Diagnostic Interview- Revised (ADI-R) and Autism Diagnostic Observation Schedule (ADOS). Over the course of 4 days of autism summer camp spread over 2 years 30 TD and 25 ASD participants were retained for the inhibitory control analysis. See Tables 1, 2 for information on participant demographics. Individuals with an IQ less than 70 were excluded from the study. Participants with fewer than 30 correct nogo trials, or with a d-prime score less than 0.5 were also excluded from the analysis (Cohen and Polich, 1997; Duncan et al., 2009). D-prime scores incorporate standard deviation or noise distribution, hit rate, and false alarm rate in its formula for calculating overall response accuracy. Finally, due to the high inter-individual variability observed in the superimposed ERPs of individual subjects, significant outliers, based on mean amplitude readings of 1.5 × the interquartile range for the N200 and P300 peaks were also excluded (Leong and Austin, 2006). Outliers also present a risk of transforming the data into a non-gaussian distribution, therefore, for a more robust measure, they were rejected (Krauledat et al., 2007). From the remaining participants, only extreme outliers, as characterized by mean amplitude readings of 3 × the interquartile range, were removed for the N170 analysis in order to retain a maximal participant count.

**Table 1:** Participant demographics for analysis of the N200, P300, and N170 component amplitudes and latencies.

ERP component at electrode location	Group	N	Sex (number of female participants)	Age	IQ (WASI-II)	Comorbid ADHD
N200/P300 at Cz and/or Pz	TD	30	9	9.6 ± 1.8	107 ± 10	0
	ASD	25	5	10.0 ± 2.0	104 ± 18	6
N170 at P3	TD	30	9	9.6 ± 1.8	107 ± 10	0
	ASD	19	3	10.1 ± 2.0	108 ± 17	5
N170 at P4	TD	30	9	9.6 ± 1.8	107 ± 10	0
	ASD	24	5	10.0 ± 2.0	105 ± 17	6

*The table below shows group comparisons of sex, age, IQ and comorbid-ADHD across groups.*

**Table 2:** Mean and standard deviation reports of accuracy, response times and trial count of artifact-free go and nogo trials across groups (ASD, TD).

Dependent variable	TD						ASD					
	RT		Accuracy		Trial count		RT		Accuracy		Trial count	
	*M*	*SD*	*M*	*SD*	*M*	*SD*	*M*	*SD*	*M*	*SD*	*M*	*SD*
Go trials	405.8	52.1	89.5	9.7	234.4	47.1	405.1	99.2	86.7	11.5	221.0	52.8
Nogo trials	–	–	74.1	11.9	66.1	16.5	–	–	69.3	15.9	57.3	17.9

Participants were between the ages of 6 and 12 years, and no significant group differences were identified for age, sex or IQ. For the N170 component analysis, six participants were removed at electrode P3 in the ASD group, since this site was used as an electro-oculogram (EOG) electrode. The following analysis employs a representative sample of the ASD population such that ASD participants with a comorbid attention deficit hyperactivity disorder (ADHD) diagnosis are included in the data analysis (Tye et al., 2014a).

## Ethics Statement

This study was carried out in accordance with the recommendations of the human research ethics guidelines from the Simon Fraser University (SFU) Office of Research Ethics. Written informed consent in accordance with the Declaration of Helsinki was obtained from each parent/guardian and written informed assent was obtained for each participant. The protocol was approved by the office of research ethics at SFU.

### Inhibitory Control Task

**FIGURE 1:**
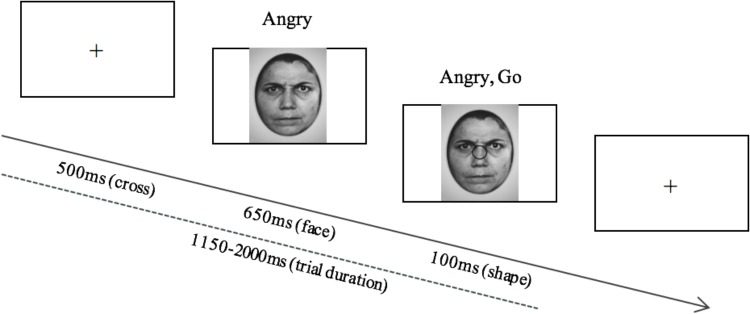
The stimulus display and its time course shown for the go/nogo task displaying the angry, go condition. After the presentation of the fixation cross, an angry or happy face is presented, followed by a circle or a square, to which the participant is either required to respond (circle) or inhibit a response (square).

EEG measurements were recorded during a computerized emotional go/nogo inhibitory response task as depicted in Figure 1. Emotional faces (happy or angry) were presented in the center of a computer screen followed by the presentation of a shape (circle or square). Both shapes and faces were randomized. Participants were instructed to ignore the faces, and to press the space bar when they observed a circle on the screen, and to not respond when they observed a square. Squares appeared in 20% of the trials, and circles appeared in the other 80% of the trials. Angry and happy faces each appeared 50% of the time. The Ekman-face stimuli were black and white and were 11 cm × 15 cm in size, and the approximate distance from the participant’s eyes to the monitor was 75 cm.

Participants performed the task in increments of 100 trials; receiving 60 s breaks between every 100 trials. A maximum of 500 trials per participant were collected throughout the task. The maximum task duration was 20 min and 40 s. Prior to task presentation, experimenters read from a script containing the task instructions, followed by a brief training period, where participants were able to practice responding to stimuli (10 stimuli during the practice session). Participants were instructed to respond to the shapes as accurately as possible, however, they were not given any instruction on preferred speed of response. Participants were expected to achieve at least 80% accuracy on the practice trials before beginning the task.

### EEG and Task Performance Data Acquisition

The EEG data were recorded using 8-channel g.Nautilus EEG systems (manufactured by g.Tec Medical Engineering) at the SFU Behavioral and Cognitive Neuroscience Institute (BCNI). The g.Nautilus system was chosen due to its comfort for the children, quick application, and excellent signal quality (Ghosh Hajra et al., 2016; Radüntz, 2018). ERPs were recorded from electrodes Fz, Cz, Pz, P3, and P4 at a sampling rate of 500 Hz. The EOG was monitored with two electrodes placed above and beside the left eye. A ground electrode placed on the forehead, and a reference electrode placed on the right ear lobe were also used. Prior to behavioral task administration, a resting state EEG measurement was recorded over a period of 3 min. During this recording, the participant was asked to sit still while focusing on a fixation cross. Accuracy and reaction time were recorded in addition to electrophysiological data during task performance.

### Behavioral Measures

Prior to, and during, experimental testing at the autism summer camp, parents of the children attending the camp were asked to complete a series of questionnaires, including the Autism Quotient (AQ), the Behavior Rating Inventory of Executive Function (BRIEF-2), the Behavior Assessment System for Children (BASC-2) and the Multidimensional Social Competence Scale (MSCS). During the summer camp the child participants also completed the Wechsler Abbreviated Scale of Intelligence (WASI-II) Intelligence Quotient (IQ) with a researcher in a separate room. See Supplementary Appendix for a detailed description of each questionnaire and the WASI-II examination.

### Data Analysis

All data analyses were performed using SPSS, Matlab, and the open-source Fieldtrip toolbox. A *p*-value less than 0.05 is received as statistically significant in the following analyses. Electrophysiological results for the inhibitory control analysis (N200 and P300 amplitude and latency) were corrected for multiple comparisons. After Bonferroni correction, the null hypothesis was rejected if the *p*-value for this analysis was less than 0.0125. The PLS analysis correlating cognitive indices with N200 amplitude uses permutation and bootstrapping methods in a single test. Accordingly, the global PLS test does not require correction for multiple comparisons.

### EEG Data Analysis

Preprocessing of the EEG data was performed with a 0.5–25 Hz, 4th order Butterworth bandpass filter (Tanner et al., 2015). Trial epochs of 200 ms before the onset of the stimulus to 800 ms after the onset of the stimulus were obtained. For analysis of the N200 and P300 components, these epochs were locked to the onset of the go/nogo stimulus. For the N170 component analysis epochs were locked to the onset of the face stimulus. Trials with significant eye movements and eye blinks were rejected based on a *z*-value cutoff of 6 obtained from average EOG. Trials containing components with peak amplitudes greater than 150 μV or less than −150 μV in the EEG channels Fz, Cz, Pz, P3, and P4 were also rejected. Stimulus-locked group average ERP’s were calculated for trials on which participants responded correctly, with a 200 ms pre-stimulus baseline correction (absolute).

Mean amplitudes of the N200, P300, and N170 components were calculated from electrode and latency windows obtained from current literature and/or visual analysis of grouped average component latency onset. The Cz electrode was used to measure mean amplitude and latencies of the N200-go, N200-nogo, and P300-nogo components, while the Pz electrode was used to measure the mean amplitude and latency of the P300-go component (Bokura et al., 2001; Jonkman et al., 2003; Jonkman, 2006; Sokhadze et al., 2009). N170 component amplitudes and latencies were calculated from an average of all trials, as well as all happy (N170-happy) and angry (N170-angry) trials separately for each participant at electrodes P3 and P4. The selection of P3 and P4 electrode locations were necessitated by the equipment limitations, however, typically posterior temporal-occipital electrode locations are used in such an analysis (Batty and Taylor, 2003). Based on the extant literature and visual analysis of the individual and group averaged ERP’s, the latency windows of maximal amplitude that were used to calculate the mean amplitude of a given component were 300–400 ms for the N200-go/nogo components, 450–600 ms for the P300-nogo component, and 450–600 ms for the P300-go component (Johnstone et al., 2005; Espinet et al., 2012; Vuillier et al., 2016). N170 latency windows of maximal amplitude were calculated at 220–320 ms (Taylor et al., 1999). Peak latency values were obtained by selecting the specific time at which the maximal amplitude occurred within the latency window of interest for each component.

The go and nogo N200 and P300 ERP component amplitudes and latencies, as well as the happy and angry N170 component amplitudes and latencies, were analyzed at both within-subject and between-subject levels using mixed-model repeated measures analysis of variance (RM-ANOVA). More specifically, mean go and nogo N200 and P300 component amplitudes and peak latencies were utilized in a RM-ANOVA analysis with Inhibition, being the type of inhibitory stimuli (go, nogo), as the within-subject factor, and Group (TD, ASD) as the between-subject factor. For the N170 analysis, a RM-ANOVA was employed for both N170 amplitudes and latencies with Face (angry, happy) and Location (P3, P4) as the within-subject factors and Group (TD, ASD) as the between-subject factor. *Post hoc t*-tests were applied for any significant results obtained in the RM-ANOVA analyses.

### Behavioral Data Analysis

The behavioral task responses (average accuracy and reaction times) of the ASD group and the TD group were calculated and presented as d-prime scores. A RM-ANOVA of go and nogo accuracies was implemented with Inhibition (go/nogo accuracies) as the within-subject factor, and Group (TD, ASD) as the between-subject factor. *Post hoc t*-tests were applied for any significant results obtained in the RM-ANOVA analyses. Differences between angry and happy go and nogo trial accuracy and reaction times were also calculated using a paired-samples *t*-test, and independent samples *t*-test for both within-group and between-group analyses, respectively.

### Correlation Analysis

Relationships between age, ERP amplitudes and latencies and behavioral responses including accuracy and reaction time were quantified for both the ASD and TD groups with Pearson correlations. Associations between significantly differing neural responses across groups and raw scores from the IQ, BRIEF-2, AQ, BASC-2, and MSCS measures for both the ASD and TD groups were ascertained using a behavioral Partial Least Squares (PLS) analysis (McIntosh et al., 1996). Behavioral PLS is a multivariate technique used to assess the statistical reliability of potential associations between neurological responses, in this case ERP amplitudes and latencies, and another matrix of behavioral or psychometric variables. Linear combinations of the original brain and behavior variables are called latent variables (LV) and the singular value associated with each pair of LV’s obtained from brain and behavior data reflect the covariance between these variables.

In the current study, our behavioral PLS was based on 10,000 permutations for the global test, which produces a *p*-value for each LV, and 10,000 bootstrap measurements for the local tests, which produce z-score’s for each individual neural score (in our case, the N200-go and N200-nogo amplitudes), indicating the strength and contribution of these scores to the overall brain-behavior associations. Standard errors, reflecting signal reliability, are estimated through the bootstrapping procedure, whereas the differentiation of signal from noise is detected through permutation calculations (Krishnan et al., 2011).

## Results

### Behavioral Results

Accuracy results from the RM-ANOVA analysis reveal a trend toward a Group main effect [*F*(1, 53) = 3.662, *p* = 0.061, η*_p_*^2^ = 0.065], however, upon a *post hoc* independent samples *t*-test analysis, no significant differences were found between groups on both the go and the nogo accuracies (Table 2). An independent samples *t*-test showed a trend toward group differences of the d-prime score [*t*(53) = 1.801, *p* = 0.077], suggesting more accurate responses in the TD group (*d’* = 2.13) compared to the ASD group (*d’* = 1.83). An independent samples *t*-test of reaction time showed no significant differences across groups. Additionally, no between-group or within-group differences were found when comparing accuracies and reaction times of both happy and angry go and nogo trials (happy-go, angry-go, happy-nogo, angry-nogo).

### EEG Results

#### N200 Responses to Go/Nogo Stimuli

**FIGURE 2:**
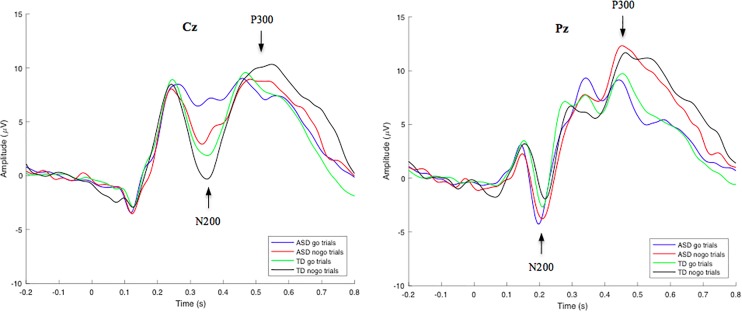
Grand-average go/nogo stimulus-locked waveforms for correct go and nogo trials in TD and ASD groups at electrode site Cz (left) and Pz (right). Mean N200 and P300 component amplitudes were obtained from latency windows of 300–400 ms and 450–600 ms, respectively. N200-go, N200-nogo, and P300-nogo amplitudes were measured at electrode Cz, while P300-go amplitude was measured at electrode Pz.

Group-averaged waveforms, calculated by the mean of individual subject data, are shown in Figure 2 for the N200 and P300 components. Using a RM-ANOVA, a main Inhibition effect was identified [*F*(1, 53) = 29.820, *p* < 0.001, η*_p_*^2^ = 0.360] at electrode site Cz, indicating a larger N200 amplitude on nogo trials compared to go trials in both the ASD (2.96 μV difference) and TD (2.22 μV difference) groups. No Inhibition × Group interaction was identified, indicating no significant variance between go/nogo amplitude differences across groups. However, a main Group effect was found [*F*(1, 53) = 6.939, *p* = 0.011, η*_p_*^2^ = 0.116], indicating differences in the go and/or nogo N200 amplitudes across groups. A *post hoc t*-test analysis revealed that both the N200-nogo and N200-go peaks were significantly more negative in the TD group compared to the ASD group [*go*: *t*(53) = −2.950, *p* = 0.005; *nogo*: *t*(53) = −2.041, *p* = 0.046]. No significant within group or between group differences were identified for N200 go and nogo latencies.

#### P300 Responses to Go/Nogo Stimuli

Through a RM-ANOVA analysis, a main effect of Inhibition was identified for the P300 component [*F*(1, 53) = 12.326, *p* < 0.001, η*_p_*^2^ = 0.189], revealing greater P300-nogo amplitudes at electrode site Cz, compared to P300-go amplitudes at electrode site Pz in both the ASD (1.97 μV difference) and TD (2.39 μV difference) groups. No other significant interactions or main effects were identified for the P300 amplitude. However, a main Inhibition effect was identified for P300 latency [*F*(1, 53) = 23.144, *p* < 0.001, η*_p_*^2^ = 0.304], revealing longer P300-nogo latencies at electrode site Cz, compared to P300-go latencies at electrode site Pz in both the ASD (33.7 ms difference) and TD (45.1 ms difference) groups. No Inhibition x Group interaction was identified for the go/nogo P300 latencies.

#### N170 Responses to Emotional Face Stimuli

**FIGURE 3:**
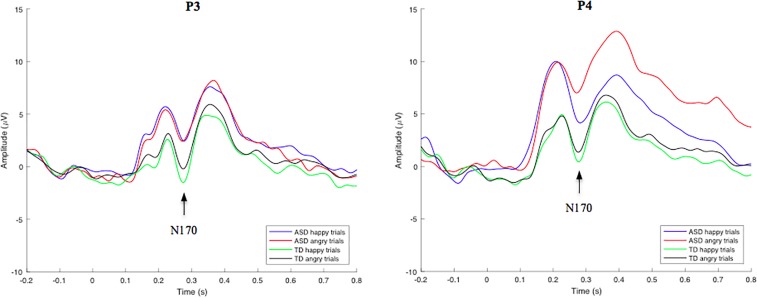
Grand-average face stimulus-locked waveforms for happy and angry trials in TD and ASD groups at electrode site P3 (left) and P4 (right). Mean N170 component amplitudes were obtained from latency windows of 220–320 ms at both electrodes.

Group-averaged waveforms, calculated by the mean of individual subject data, are shown in Figure 3 for the N170 component. In an independent samples *t*-test employing an average of all trials for each participant, no significant overall N170 amplitude or latency differences were identified between groups. N170-happy and N170-angry ERP’s were also calculated for both the ASD and TD groups at electrode location P3 and P4. Using a RM-ANOVA, results showed no main Face effect, suggesting that there were no significant differences in the neural responses to angry compared to happy faces across all subjects. There was also no main Group effect, showing no significant differences between groups on N170 amplitudes and latencies during both angry and happy face processing. However, interestingly, a Location × Face × Group effect was identified [*F*(1, 53) = 6.342, *p* = 0.015, η*_p_*^2^ = 0.119] for N170 amplitude, revealing that the ASD group showed a larger difference between happy and angry trials than the TD group; an effect that was particularly pronounced at the P4 electrode compared to the P3 electrode. No other interaction effects were identified. Since no behavioral differences were found for angry vs. happy effect on inhibitory control response accuracy, and due to low trial numbers, the comparative effect of angry and happy faces on go and nogo neural responses was not analyzed.

### Age, N200 and D-Prime Correlations

Given the significant between group differences in the N200 component amplitude, this variable was selected for analysis of correlations with behavior. Significant associations between age, d-prime, and N200 ERP amplitude were observed between each measure in the TD group. The results show that as age increases, N200-go (*P* < 0.01) and N200-nogo (*P* < 0.05) amplitudes decrease, and d-prime scores increase (*p* < 0.01). However, only correlations between age and d-prime (*p* < 0.05), and age and N200-go scores (*p* < 0.05) were identified in the ASD group.

### Correlations Between Electrophysiology and Parent Rating Scales of Behavior

**FIGURE 4:**
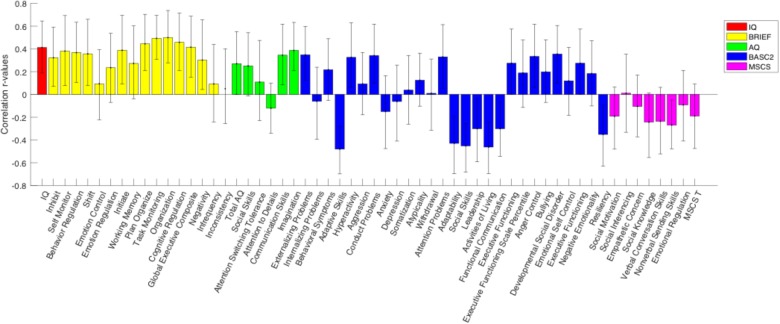
PLS analysis between N200 amplitude and behavioral scores for the TD group. The height of the correlation bars indicates the magnitude of the *r*-values representing the correlation between behavioral measures and neural data, while the direction of the correlation bars indicates the type of association between behavioral measures and neural data. A positive bar signifies a positive correlation between behavioral measures and neural scores (i.e., as the behavioral score increases, N200 voltage also increases). Error bars reveal an upper and lower error range for the correlation between behavioral measures and neural data based on a series of bootstrapping analyses. An error bar that does not include zero indicates a significant correlation between the behavioral measure and N200 amplitude.

A behavioral PLS analysis was performed to test for significant associations between the N200 component amplitude and the IQ, BRIEF-2, AQ, BASC-2, and MSCS scores, separately for each group. A significant overall correlation between N200 amplitude and all behavioral scores was identified in the TD group (*p* = 0.048), however, no significant overall correlation was found in the ASD group. Figure 4 illustrates the correlations between behavioral test subscores and N200 amplitude in the TD group, alongside their error bars, which reveal an upper and lower error range for the correlation based on a series of bootstrapping analyses. Given that a high number of error bars in the BRIEF-2 subscores that do not cross zero, the significant correlation between N200 amplitude and behavioral scores in the TD group appears to be driven largely by the BRIEF-2 subscores. The subscores of the AQ, given their relatively small error bars, also evidently drive this correlation. Overall, these results show that increased N200 component amplitude is associated with improved executive function and fewer autism traits.

## Discussion

The present study shows reduced neural responses related to conflict monitoring in children with ASD compared to TD children, evidenced by reduced N200 amplitudes during an emotional go/nogo task. Furthermore, this is the first study to show significant correlations between N200 amplitude, age, d-prime scores, and various behavioral scores in TD children alongside no such correlations in children with ASD, possibly indicating differences in neurophysiological development related to inhibitory control. The results from the TD population suggest that N200 component amplitude, elicited by a go/nogo inhibitory control task, is an indicator of functional inhibition deficits and is modulated by neural development.

### Neurophysiological Responses to Go/Nogo Stimuli

The reduced N200-go and N200-nogo component amplitudes in the ASD group suggest that individuals with ASD experience possible abnormalities with later-stage differentiation of stimuli (i.e., conflict monitoring) and response decision-making processing. Electrophysiological differences related to inhibitory control in the absence of hypothesized task performance differences may indicate that measurements such as N200 amplitude are more sensitive markers for inhibitory control deficits in ASD than behavioral measures in this age range (6–12). Alternatively, it is possible that the lack of task performance differences may indicate that there are no functional inhibition differences between children with and without ASD. These results are supported by previous findings, showing an absence of behavioral differences paired with significant neurophysiological differences during an inhibitory control task in individuals with ASD compared to TD individuals (Larson et al., 2012).

Using functional magnetic resonance imaging, a study by Kana et al. showed that during an inhibitory control task, ASD participants revealed less brain activation in areas responsible for inhibition, including the ACC, compared to typically developing individuals (2007). The ACC is consistently identified as the neural generator of the N200 component reflecting conflict monitoring (Van Veen and Carter, 2002; Bekker et al., 2005; Clayson and Larson, 2011). The current results of decreased N200 amplitude in the ASD group may be consistent with findings such as the one shown by Kana et al., and thus also support the theory that decreased brain activation in individuals with ASD may be correlated with less automatic inhibitory control mechanisms. Overall, there was only a trend toward group differences of the d-prime score, while significant neural response differences between groups were identified, suggesting that children with ASD might utilize compensatory or less-specified neural networks to achieve similar behavior results compared to TD individuals.

No differences in the go and/or nogo P300 component amplitude were identified between groups, suggesting no differences across groups on the cognitive processes related to the actual inhibition of the motor response. These unexpected results may suggest that children with ASD do not differ significantly in cognitive processes related to the actual inhibition of the motor response on a go/nogo task compared to TD individuals. These results do not align with our initial hypothesis, which was driven by a study assessing children of 5 years of age (Kim et al., 2018). This is possibly due to developmental factors, such that at an older age, individuals with ASD are more able to inhibit a motor response (2017). However, an effect of inhibition was identified for both the N200 and P300 amplitude. An Inhibition effect for latency was only identified for the P300, revealing longer latencies for the nogo condition compared to the go condition across all participants. This may suggest that the increased difficulty of inhibiting a prepotent response compared to continuing a prepotent response, requires more effortful processing, resulting in a slower neural response during nogo trials. In the absence of a Group x Inhibition effect for N200 and P300 component latency, the data also suggest that both children with and without ASD process nogo stimuli at a similar speed.

### Neurophysiological Responses to Face Stimuli

Abnormal face processing strategies in individuals with ASD have been previously reported (Dawson et al., 2005). In the current study, however, no significant differences in N170 amplitude or latency in response to emotional face stimuli were identified between TD and ASD groups. Additional analysis showed no statistically significant amplitude or latency differences across groups for both the N170-happy and N170-angry stimuli. However, a Location x Face x Group effect was identified for N170 amplitude, revealing that at the P4 electrode site, the ASD group showed a larger difference between happy and angry trials compared to the TD group, indicating potential atypical lateralized response to automatic emotion processing in these individuals. No follow up analyses were performed on this 3-way interaction because (a) this interaction effect was not an *a priori* hypothesis (b) this study had a relatively small sample size, and (c) this study had inadequate electrode locations/count needed for a proper lateralization analysis. Overall, this study is underpowered to perform such analyses, therefore, to prevent presenting misleading information, no follow up analyses were performed on this 3-way interaction. Given the opposing findings of various other research groups, and the seemingly significant differences of N170 amplitude across groups, as observed in Figure 3, it is possible that these findings are reflective of a smaller sample size, and larger sample studies are necessary to resolve this dichotomy.

Multiple studies have shown that emotional stimuli can have negative effects on performance of an inhibitory control task, suggesting that emotional stimuli interrupt ongoing cognitively-controlled tasks, such as the inhibitory control task, ultimately impairing task performance (Verbruggen and De Houwer, 2007; de Houwer and Tibboel, 2010; Kalanthroff et al., 2013). However, at a behavior level, no statistically significant differences of accuracy or reaction time for the happy vs. angry go and nogo trials were identified for both the ASD and TD groups.

In summary, these results suggest that individuals with ASD (a) do not possess significant impairments on automatic emotion processing of happy and angry faces at a neural level compared to TD individuals, (b) do not show accuracy differences in inhibitory control in happy vs. angry conditions, and (c) show no main differences in inhibitory response accuracy for both happy and angry conditions compared to TD individuals. Overall, these results show that individuals with ASD process automatic emotion stimuli no differently than TD individuals, and therefore, it can be assumed that no significant differences in the interaction effect between face stimuli and go/nogo task performance or neural processing would exist across groups.

### Response Accuracy, ERP Component Amplitudes, and Age Correlations

Given the age range of all participants (6–12 years old) and the key period in development that this age range represents, it is important to identify age-related changes in neural and behavioral inhibitory responses for both ASD and TD groups. Consistent with literature, analysis of data from the TD group showed that increased N200 component amplitude was correlated with decreased task performance and younger age (Johnstone et al., 2005). This supports the theory that increasing age results in the development of more efficient cognitive control processes, which require more specified and fewer neural resources. In addition to the age and N200 correlations identified in the TD group, the ASD group also showed that as age increased N200-go amplitude decreased, possibly leading to the counterintuitive interpretation that at a young age, ASD reflects a more mature neural system than is observed in TD children. However, age and N200-nogo correlations were not significant in the ASD group, and d-prime scores and N200 amplitude relationships were also not significant in the ASD group. These findings, paired with the trend toward a group difference in task performance between groups, where children with ASD performed worse than that TD group, suggest that a more mature neural system in the ASD group is unlikely. Instead, it is possible that this reduced N200 in young children with ASD is reflective of increased variability within and/or across participants or reduced neural recruitment specific to conflict monitoring. Conversely, the correlation between d-prime scores and N200 amplitude in the TD group reveals an effective relationship between efficient/focal neural activation and improved motor response accuracy.

Given that (a) no significant relationship was identified between d-prime scores and N200 amplitude in the ASD group, (b) a trend toward a group difference in task performance was present, and (c) decreased N200 amplitude was identified in the ASD group compared to the TD group, it appears that children with ASD were less able to use enhanced or more focal ACC-driven processing to moderately improve their response accuracy compared to TD individuals.

### Neurophysiological and Behavioral Measures

The results showed an overall significant correlation between N200 component amplitude and multiple behavioral scores/subscores in the TD group. Specifically, results showed that as N200 amplitude increases, executive function abilities, as measured by the BRIEF-2 assessment, improve. This is not surprising, since BRIEF-2 scores are adjusted for age, and therefore, as amplitude increases at a young age (more ideal/specified neural development), their executive function abilities are also enhanced relative to young children with less ideal neural functioning (reduced N200 component amplitude). The results also indicate a reliable correlation between AQ and N200 amplitude in the TD group, signifying that as autism traits increase, N200 amplitude decreases, giving further support to the proposal that decreased N200 amplitude is reflective of ASD symptomology at a young age.

No overall brain-behavior association was identified in the ASD group, which may be reflective of a small sample size, and may, therefore, reflect a study limitation rather than a meaningful brain-behavior abnormality in children with ASD. Additionally, despite the non-motoric nature of the N200 component making it relatively optimal for correlations with other behavioral measures, it is difficult to directly compare N200 component amplitudes with various behavioral measures, since the N200 component is generated in regions of the prefrontal cortex, and during specific tasks such as the go/nogo task, the generators become more specified. The scores indicated in the behavioral measures do not reflect specific brain regions in the same way, again making direct comparisons difficult. However, the results indicated here are the first to identify associations between neurophysiological responses of inhibitory control and the listed behavioral scores in ASD and TD children.

### Limitations

Due to the high number of subjects with comorbid ADHD, one of the main limitations of the current study is that we did not analyze the neural correlates of emotion processing and inhibitory control in a strictly ASD population, and therefore, the results may be influenced by other tendencies of ADHD, including impulsivity. However, previous research by Tye et al. (2014a) showed that individuals with ASD without comorbid ADHD show reduced N200 amplitude to an inhibitory control task, whereas individuals with ADHD show reduced P300 amplitude to an inhibitory control task. Therefore, the reduced N200 component amplitude appears to be reflective of neurophysiological differences relating to inhibitory control in ASD rather than ADHD. Additionally, we ran a subsequent analysis to determine whether the participants with comorbid ADHD were driving the observed effects. No alternate findings were identified when the participants with comorbid ADHD were removed from the analyses.

Our hypotheses of reduced N170 amplitude and prolonged latency, as well as subsequent associations between abnormal N200, P300, and N170 component amplitudes and latencies with various behavior scores and age were largely not supported. One main limitation potentially lending to these findings is that there were relatively few participants after meeting our exclusion criteria. Ideally, to draw more accurate conclusions regarding developmental neural trajectories, and behavioral-neural correlations, one would need to employ a larger sample size. Additionally, for the N170 analysis, more temporal-occipital electrode locations (Batty and Taylor, 2003) would likely show a more accurate representation of emotion processing abilities and possible differences across groups.

Although the N200 and P300 are the two most commonly reported ERP components elicited in a go/nogo task, other components such as prefrontal N100, P100 and P200 are also consistently elicited during such a task (Berchicci et al., 2016; Sulpizio et al., 2017). However, these prefrontal components are typically localized to the Fz electrode site, and six of the participants in the ASD group in the current study were tested with an EEG cap that had a broken Fz electrode. Therefore, although it would be valuable to analyze these components, our useable electrode locations did not permit an adequate analysis of such components. Future studies should seek to identify possible differences in exogenous cognitive processing across children with ASD and TD children.

Lastly, our group did not administer a go/nogo task without the presence of faces behind the shapes. This made it impossible to directly compare the effect of face processing on N200 and P300 component amplitudes and latencies. Similarly, our paradigm was not designed to compare the neurophysiological responses to emotional stimuli with non-social control stimuli. Future studies should employ a task that is designed to address these aims.

## Conclusion

By analyzing EEG measurements of inhibitory control, along with age and behavioral assessments, we provide the first evidence of relations between neural processing relating to inhibitory control and particular cognitive and affective consequences during development in TD children alongside no such relationships in children with ASD. Children with ASD also showed reduced N200 component amplitude scores compared to TD children during the emotional go/nogo task. Consequently, our findings provide new evidence for differences in neurophysiological responses relating to inhibitory control in children with ASD compared to TD children. EEG is, in relative terms, a low-cost method of tracking the brain’s neurophysiological changes, and alongside behavioral and parent rating assessments, could prove to be a useful and objective assessment tool for clinicians and therapists to utilize once a supported outline of characteristic neurophysiological outputs for ASD and TD individuals is determined. Acquiring data in an atmosphere such as the one utilized in this study, where all data collection was performed in a single room with trained undergraduate/graduate volunteers, provides meaningful translational information for such clinical or school-based assessments/treatment efforts.

## Data Availability

The datasets generated for this study are available on request to the corresponding author.

## Author Contributions

JM, NP, SF, RD, GI, SM, and SD contributed to conception and design of the study. JM, NP, AN, GC, SM, and SD contributed to acquisition and analysis of data. SF, GI, RD, and UR contributed to acquisition of data. VV contributed to analysis of data. JM wrote the first draft of the manuscript. All authors contributed to manuscript revision, read, and approved the submitted version.

## Conflict of Interest Statement

NP was employed by company CTF MEG International Services. The remaining authors declare that the research was conducted in the absence of any commercial or financial relationships that could be construed as a potential conflict of interest.
