# Supplementary material for: Electrophysiology of Inhibitory Control in the Context of Emotion Processing in Children With Autism Spectrum Disorder
Source: Front Hum Neurosci. 2019 Mar 12;13:78. doi: 10.3389/fnhum.2019.00078 (PMC6422887; doi:10.3389/fnhum.2019.00078)
Supplement: Supplementary file 1 [file Data_Sheet_1.docx]

**WASI-II:**

The WASI-II examination assesses the intelligence of examinees aged 6 through 90 years. This test includes both perceptual reasoning abilities (block design and matrix reasoning) and verbal intelligence (vocabulary and similarities). The WASI-II is often used for research purposes to match experimental groups based on cognitive abilities, with higher scores indicating higher intelligence.

**AQ:**

The 50-item AQ parent-report is used to quantify autism traits including social skills, communication skills, attention to detail, imagination and attention switching/tolerance of change in children 4-11 years old (Auyeung et al., 2008). The AQ is assessed on a scale from 0 to 50, with higher overall scores signifying higher levels of autism traits.

**BRIEF-2:**

The BRIEF-2 parent-form is used to measure executive function in children aged 5 to 18 years old (Gioia et al., 2000). Executive function is measured by two main categories: behavior regulation and metacognition. Behavior Regulation is divided into three subgroups, which include i) Inhibit (the ability to control impulses/behaviors), ii) Shift (the ability to tolerate change or alternate attention), and iii) Emotional Control (the ability to regulate emotional responses). The Metacognition index is divided into five categories, which include i) Initiate (the ability to start an activity and develop ideas/problem solving strategies related to the activity), ii) Working Memory (the ability to retain information during task completion or when encoding information), iii) Plan/Organize (the ability to set goals, retrieve main points in presentations, develop steps, etc.), iv) Organization of Materials (the ability to create order in one’s workspace, such as their desk or backpack), and v) Monitor (the ability to assess previous work and determine performance level, as well as the ability to reflect on how one’s behavior has affected others) (Gioia et al., 2000). Higher scores indicate greater executive function impairments.

**BASC-2:**

The BASC-2 parent rating scale yields primary clinical, adaptive, and content scores, which are used to determine composite scores of externalizing problems, internalizing problems, behavioural symptoms and adaptive skills in children/adolescents aged 2-21 (Flanagan, 1995). Primary clinical scales include aggression, anxiety, attention problems, atypicality, conduct problems, depression, hyperactivity, somatization and withdrawal. Primary adaptive scales include adaptability, functional communication, leadership and social skills. And finally, content scales include anger control, bullying, developmental social disorders, emotional self-control, executive functioning, negative emotionality, and resiliency. Higher scores indicate greater impairment on every scale other than the adaptive functioning scales.

**MSCS:**

Lastly, the MSCS is a scale assessing the social competence of an individual, and is split into seven domains, which include social motivation, social inferencing, demonstrating empathetic concern, social knowledge (understanding relationships and social context), verbal conversation skills, nonverbal sending skills (sending non-verbal communication including eye contact, facial expressions, tone of voice, gestures, etc.) and emotion regulation (Yager and Iarocci, 2013). The MSCS was designed with the aim of assessing social behaviours commonly identified among the high functioning ASD population, and sometimes identified in TD individuals presenting milder levels of social impairment (Yager and Iarocci, 2013). Higher scores on the MSCS reflect higher levels of social competence (Yager and Iarocci, 2013).
